# Is the Diagnosis of Celiac Disease Possible Without Intestinal Biopsy?

**DOI:** 10.4274/balkanmedj.2016.1258

**Published:** 2017-08-04

**Authors:** Maha Shomaf, Mohammad Rashid, Dana Faydi, Ahmad Halawa

**Affiliations:** 1 Department of Pathology, Jordan University School of Medicine, Amman, Jordan; 2 Department of Internal Medicine, Jordan University School of Medicine, Amman, Jordan; 3 Jordan University School of Medicine, Amman, Jordan

**Keywords:** Coeliac disease, intestinal biopsy, tissue transglutaminase

## Abstract

**Background:**

Coeliac disease is defined as a state of immune-mediated hyper-responsiveness to dietary gluten from wheat, barley, or rye in genetically predisposed individuals that results in tissue damage. The diagnosis is made by microscopic examination of a small intestinal biopsy, although serological testing for antibodies against tissue transglutaminase and deamidated gliadin peptide can be of great advantage. It has been suggested that duodenal biopsy can be avoided in patients with high levels of the tissue transglutaminase antibody, since a relationship has been found to be present between tissue transglutaminase antibody titres and coeliac disease.

**Aims:**

To study the correlation between tissue transglutaminase titre and small intestinal biopsy findings in patients with coeliac disease.

**Study Design:**

Diagnostic accuracy study.

**Methods:**

Ninety-five cases of patients diagnosed with coeliac disease and with positive serum tissue transglutaminase titres were retrieved from the Jordan University Hospital archives between December 2014 and December 2015. All the cases were classified according to the Marsh classification.

**Results:**

Ninety-five cases with a positive titre for the antibody were included in this study, 73 (76.8%) of them were females and 22 cases (23.2%) were males. The age of the patients ranged between 4 and 75 years with a mean age ± standard deviation of 32.3±14.7. The sensitivity was the highest in Marsh IIIC and lowest in Marsh IIIA (95% versus 68% respectively). The specificity was moderate (76%) for all subtypes of Marsh III.

**Conclusion:**

This study showed a positive correlation between the tissue transglutaminase titre and the degree of duodenal damage (Marsh IIIC) in patients with coeliac disease. In the presence of high tissue transglutaminase levels, duodenal biopsy might not be always necessary for diagnosis, particularly in symptomatic patients.

Coeliac disease is defined as a state of immunologic hyper-responsiveness to dietary gluten from wheat, barley, or rye in genetically predisposed individuals that results in tissue damage (1). It affects approximately 0.5-1% of the Western population (2).

The incidence of coeliac disease is increasing worldwide, due to increased recognition of the variability in its clinical presentation. Since the fifties, the diagnosis of coeliac disease has depended on duodenal biopsy findings that include different degrees of villous atrophy along with crypt hyperplasia and intraepithelial lymphocytosis, which are graded by the Marsh classification (Marsh I-IIIC) (3,4). The development of highly sensitive serological tests such as anti-tissue transglutaminase (tTG autoantibodies), anti-endomysial antibody (EMA) and anti-deamidated gliadin peptides (anti-DGP antibodies) have facilitated the diagnosis of coeliac disease, particularly in asymptomatic patients or in patients with vague clinical presentations (5,6).

In this study we used data from patients diagnosed with coeliac disease and assessed the correlation between duodenal histological Marsh grading and tTG antibody titres.

## MATERIALS AND METHODS

Ethical approval was obtained from the institutional review board of Jordan University Hospital (approval no: 10-2016/9810). Informed consent was obtained from participants with identifying information.

### Study design

This is a retrospective, single centre study carried out at Jordan University Hospital between December 2014 and December 2015. Institutional ethics committee clearance was obtained.

### Study population

One hundred and ninety-three cases with small intestinal biopsies between December 2014 and December 2015 were retrieved from the hospital archives. All of the biopsies were performed to exclude coeliac disease based on clinical suspicion. One hundred and thirty-three cases had serological titres of tTG IgA antibodies. Ninety-five of these cases had positive titres and were included in the analysis. Endoscopies and pathology reviews had been performed by experienced gastroenterologists and a single trained pathologist. Pathology reports from duodenal biopsies had been reported following the modified Marsh classification.

### Serology

All of the patients had anti-tTG IgA antibody titres. The test was performed using the enzyme-linked immunosorbent assay (ELISA) technique with a commercially available kit (generic assays, generis assays GmbH, Germany). Antibody levels above 20 IU/mL were considered positive, as per the manufacturer’s recommended tTG cut-off value.

### Statistical analysis

The collected data were analysed using SPSS® software, version 20.0 (IBM Corp. Released 2011. IBM SPSS Statistics for Windows, Version 20.0. Armonk, NY: IBM Corp). Data were summarised as mean ± standard deviation or counts (%) as appropriate.

Differences between patients with or without positive antibody titres were assessed using chi square (Marsh score and gender) and independent student t-tests (age).

Association between the standard method and the proposed alternative methods was assessed using chi-square tests. The association strength was estimated using the odds ratio (OR) and its 95% confidence interval (CI). Logistic regression was utilised when multiple variables were statistically associated with the standard method for the diagnosis of the disease.

The normality of the data was assessed using a Q-Q plot, a P-P plot and a Shapiro-Wilk test. The homogeneity of variance was evaluated using Levine's test. P values less than 0.05 were considered statistically significant.

## RESULTS

Out of 193 cases with small intestinal biopsies 133 cases had serological titres for anti-tTG IgA antibodies. Thirty-eight cases (28.6%) had negative titres, 24 (63.2%) of them females and 14 (36.8%) males (Table 1). All the cases with positive antibody titres were included in the study [95 cases (71.4%)]. Seventy-three (76.8%) of them were females and 22 cases (23.2%) were males. The age of the patients ranged between four and 75 years with a mean of 32.3±14.7.

Histopathological examination of the biopsy specimens resulted in the following Marsh scores: Marsh I - 29 cases (21.8%); Marsh II - 7 cases (5.2%); Marsh IIIA - 19 cases (14.3%); Marsh IIIB - 40 cases (30.1%); and Marsh IIIC - 38 cases (28.6%) (Table 2).

Patients with Marsh score IIIC were 6.9 years younger compared with the other grades (27.3±14.9 versus 34.2±14.3 years, p=0.014), while apparently healthy individuals (Marsh score I) were 7.1 years older than the others (37.8±14.5 versus 30.7±14.5 years, p=0.021). A similar influence of age on antibody titre was observed: recruits with positive antibody titres were six years younger than recruits with -negative titres (+ve Ab titres: 30.6±14.1, -ve Ab titres: 36.3±15.7 years, p=0.044). It should be noted that antibody positive status was independent of gender (p=0.19), which was also observed for Marsh score IIIC (p=0.38) and Marsh score I (p=0.26).

Recruits with positive antibody titres were 11 times more likely to have a Marsh score of IIIC [OR (95% CI)=11 (2.5-48.4), p=<0.0001], while recruits with negative Ab were 17.2 times more likely to have a Marsh score of I [OR (95% CI=0.06 (0.021-0.16), p=<0.0001].

To discern the true predictors of Marsh score IIIC (+ve Ab titres or age), a stepwise backward logistic regression was conducted. The positive antibody titre was the only variable retained in the final model [+ve Ab titres: OR (95% CI)=11 (2.5-48.4), p=0.0001]. The Hosmer and Lemeshow test of the model was satisfactory (p=0.17). Similar findings were obtained using stepwise forward logistic regression.

To discern the true predictors of Marsh score I (apparently healthy) (-ve Ab titre or age), stepwise backward logistc regression was conducted. The negative antibody titre was the only variable retained in the final model [-ve Ab titres: OR (95% CI)=0.06 (0.021-0.16), p<0.0001]. The Hosmer and Lemeshow test of the model was satisfactory (p=0.92). Similar findings were obtained using stepwise forward logistic regression.

Moreover, sensitivity, specificity, positive predictive value (PPV) and NPV analysis of “Ab status predicting Marsh IIIC” were encouraging. The sensitivity was highest for Marsh IIIC and lowest for Marsh IIIA (95% versus 68% respectively). The specificity was moderate (76%) for all subtypes of Marsh III (Table 3). These findings open up the possibility of using antibody titre as a new marker for the diagnosis of coeliac disease.

## DISCUSSION

In the 1980s, sensitive and specific serological tests were developed for coeliac disease. These tests are used as the first step to identify patients who should undergo intestinal biopsy analysis when the disease is suspected clinically (7). Serological markers of coeliac disease, which include IgA against tTG, EMA, IgA or IgG against DGP and IgG against tTG, have been shown to positively correlate with coeliac disease and only a small number of patients show negative results (8).

These markers have remarkably improved the diagnosis rate in screening programs for coeliac disease and have created a tendency towards using non-invasive and less expensive methods for the diagnosis of the disease, especially in children. However, this practice means there is a need to study the correlation between these serological marker levels and the mucosal damage that is seen in coeliac disease, and to determine whether it has sufficient PPV to be solely used for the diagnosis of the disease.

In 1997, Dieterich et al. (9) recognised the tTG antibody as the major endomysial autoantigen in coeliac disease. Different studies have shown that the degree of duodenal changes is correlated with tTG-IgA and EMA-IgA titres. Both antibodies have shown the highest diagnostic reliability in the majority of recent studies, with combined sensitivities of 89-90% and specificities of 98-99% (6,10). Some studies have even proposed that duodenal biopsy can even be ignored in the case of strongly positive tTG levels (11,12) and some have proposed that the presence of high antibody titres in symptomatic patients may decrease the need for intestinal biopsies to diagnose the disease (9).

In our study the sensitivity ranged between 95% and 68%, and the highest sensitivity was with Marsh IIIC cases. The specificity was moderate (76%) in comparison to Marsh IIIA and this might be attributed to the levels of antibody titres, which were higher in Marsh IIIC. Our findings are in accordance with other studies on tTG antibodies, which showed a pooled specificity of tTG-guinea pig and tTG-human recombinant between 95% and 99% in one study and 93.0% (95% CI, 91.2-94.5) in another study (11,13).

Many prospective studies have shown that the predictive value of IgA-tTG was highly significant with sensitivities and specificities greater than 90% (14,15). It appears that the diagnostic accuracy of antibody titres is related to the pre-test probability of CD with declining outcome in low-risk populations (15).

However, biopsy is still needed in patients who do not show clinical improvement on a gluten-free diet and in cases with mildly or modestly elevated IgA-tTG (16,17). The optimal cut-off point of IgA anti-tTG level to recognise patients who may not need a biopsy varies between assays. Some reports suggest a cut-off point of an IgA-tTG titre greater than 10 times the ULN, (18) while others such as Zanini et al. (10) found that titres exceeding five times the upper limit of normal (ULN) showed 100% specificity and PPV for duodenal atrophy. Alessio et al. (6) reported that all patients with IgA anti-tTG titres more than seven times the ULN showed histological features of coeliac disease. Others found that when the IgA-tTG titre is greater than 100 U/mL, biopsy is not mandatory to confirm the diagnosis, particularly in symptomatic patients (13).

The differences in sensitivity and specificity can be related to the levels of the antibodies titre and the cut-off points of the different assays used. The results of commercially available ELISA tTG assays may differ depending on different factors such as the quality of the tTG antigen (19), the method of extraction, and the processing of the antigen. All of these variables might affect the test results (20).

In addition, tTG can exist in several conformations (open, extended or closed), with variable activity of the enzyme (21), which might also be reflected in the results, with the open conformation tTG being the superior antigen (22). Thus, the different commercial tTG-ELISA tests can reveal different numbers of false-negative or false-positive results.

Laboratory testing for coeliac disease has shown steady improvement over the years, reflecting increased awareness of the pathophysiology of the disease. The correlation between molecular mechanisms and serological and genetic markers is clear, enhancing their use in clinical practice. However the current lack of standardisation between different assays and laboratories makes their use challenging. Further improvements in test accuracy are strongly required and the indications for biopsy still need to be agreed upon.

Only tTG antibody titres were studied in this study. Titres of other antibodies, such as DGP antibody, which are not done on a routine basis for our patients, would probably have an impact on the results.

In conclusion, antibody testing showed high accuracy, particularly in symptomatic patients. IgA-tTG testing should be one of the first-line tests in recently updated clinical guidelines.

## Figures and Tables

**Table 1:**
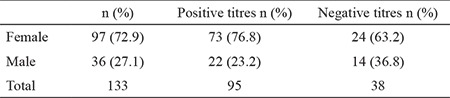
The relationship of gender with anti-tissue transglutaminase IgA antibody

**Table 2:**
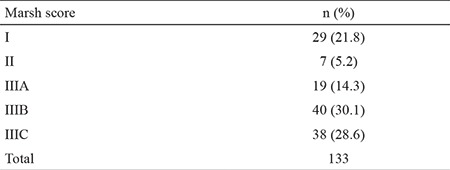
Marsh score results in the study cases

**Table 3:**
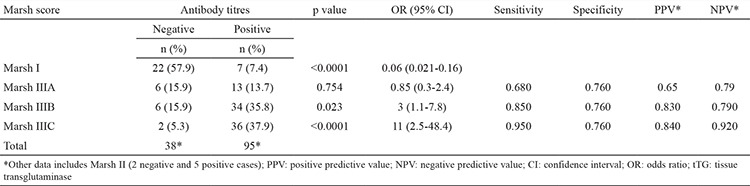
Ability of anti-tissue transglutaminase IgA antibody titre to accurately predict coeliac disease
